# Early Inflammatory Biomarkers, Ventricular Dysfunction and In-Hospital Mortality in Patients with ST-Elevation Myocardial Infarction Undergoing Primary Percutaneous Coronary Intervention

**DOI:** 10.3390/diagnostics16131978

**Published:** 2026-06-25

**Authors:** Dan Claudiu Magureanu, Maria Luiza Hiceag, Camelia Bianca Rus, Timea Claudia Ghitea, Corina Cinezan

**Affiliations:** 1 Department of Pharmacology, Toxicology and Clinical Pharmacology, Iuliu Hatieganu University of Medicine and Pharmacy, 400012 Cluj-Napoca, Romania; dan.clau.magureanu@elearn.umfcluj.ro; 2 Cardiology Department, Niculae Stancioiu Heart Institute, 400001 Cluj-Napoca, Romania; 3 Cardiology Department, Rehabilitation Hospital, 400347 Cluj-Napoca, Romania; 4 Cardiology Department, Municipal Hospital Aiud, 515200 Aiud, Romania; 5 Department of Medical Disciplines, Faculty of Medicine and Pharmacy, University of Oradea, 410073 Oradea, Romania; rus.cameliabianca@student.uoradea.ro; 6 Clinical County Emergency Hospital Bihor, 410169 Oradea, Romania; 7 Doctoral School of Biological and Biomedical Sciences, University of Oradea, 410087 Oradea, Romania; 8 Pharmacy Department, Faculty of Medicine and Pharmacy, University of Oradea, 410073 Oradea, Romania; timea.ghitea@csud.uoradea.ro

**Keywords:** ST-elevation myocardial infarction, primary percutaneous coronary intervention, inflammatory biomarkers, C-reactive protein, neutrophil-to-lymphocyte ratio, left ventricular ejection fraction, ventricular dysfunction, in-hospital mortality

## Abstract

**Background/Objectives:** Inflammation plays a central role in the pathophysiology of ST-elevation myocardial infarction (STEMI) and may influence myocardial injury, ventricular dysfunction and clinical outcomes. Simple inflammatory biomarkers derived from routine laboratory tests have been proposed as potential prognostic indicators in patients undergoing primary percutaneous coronary intervention (PCI). **Objective:** This study aimed to evaluate the association between admission inflammatory biomarkers, echocardiographic markers of ventricular dysfunction and in-hospital mortality in patients with STEMI treated with primary PCI. **Methods:** We conducted a retrospective observational study including 600 consecutive patients admitted with STEMI and treated with primary PCI between January 2021 and August 2025. Inflammatory biomarkers measured at admission included C-reactive protein (CRP); neutrophil-to-lymphocyte ratio (NLR); platelet-to-lymphocyte ratio (PLR); systemic immune-inflammation index (SII) and C-reactive protein-to-lymphocyte ratio (CLR). Echocardiographic parameters and clinical outcomes were recorded. Multivariable logistic regression analysis was performed to identify independent predictors of in-hospital mortality. **Results:** In-hospital mortality occurred in 54 patients (9.0%). Patients with reduced left ventricular ejection fraction (LVEF ≤ 40%) had significantly higher CRP and CLR levels (*p* < 0.01). Inflammatory biomarkers were associated with markers of ventricular dysfunction but were not independent predictors of mortality. Age, LVEF < 40% and the number of residual coronary lesions independently predicted in-hospital death. **Conclusions:** In STEMI patients undergoing primary PCI, early mortality is mainly determined by age; ventricular dysfunction and residual coronary disease burden, while inflammatory biomarkers primarily reflect the severity of myocardial injury rather than independently predicting short-term mortality.

## 1. Introduction

ST-elevation myocardial infarction remains a major cause of morbidity and mortality worldwide despite advances in early reperfusion strategies and contemporary pharmacological treatment. Although prompt primary percutaneous coronary intervention has significantly improved outcomes, in-hospital mortality and early complications remain substantial in high-risk patients.

Beyond ischemic burden and reperfusion delay, systemic inflammation plays a pivotal role in the pathophysiology of acute myocardial infarction. Inflammatory activation contributes to plaque development and destabilization, myocardial injury extension, adverse ventricular remodeling and multiorgan dysfunction [1,2,3]. Low-grade inflammation is a key driver not only of plaque destabilization but also of complications in acute coronary syndromes, playing a central role throughout the disease continuum [4]. In recent years, simple hematological inflammatory indices derived from routine laboratory parameters, such as the neutrophil-to-lymphocyte ratio, platelet-to-lymphocyte ratio, systemic immune-inflammation index, C-reactive protein and C-reactive protein-to-lymphocyte ratio have emerged as potential prognostic markers in acute coronary syndromes [5,6]. These low-cost, widely available markers provide independent information on risk stratification and optimal management in daily clinical practice. Elevated levels of these inflammatory indices are independently associated with increased in-hospital mortality, 30-day and 90-day mortality, major adverse cardiovascular events, stent thrombosis, arrhythmias and myocardial perfusion disorders [7]. 

Several studies have demonstrated that elevated inflammatory indices, including NLR, PLR and SII, are associated with adverse outcomes in patients with acute coronary syndromes and are linked to increased mortality rates [8,9,10]. In addition, these markers have shown prognostic value for left ventricular dysfunction and adverse cardiac remodeling following STEMI [11,12], as well as for the prediction of contrast-induced nephropathy after percutaneous coronary intervention [13,14]. Their predictive performance may be further enhanced when combined with established risk stratification tools [15,16]. However, the relationship between early inflammatory burden, left ventricular systolic dysfunction, renal impairment following primary PCI, and short-term mortality in a contemporary real-world STEMI population remains incompletely defined [17]. Therefore, the aim of this study was to evaluate whether admission inflammatory markers (NLR, PLR, SII and SIRI) are associated with reduced left ventricular ejection fraction, contrast-induced nephropathy and 30-day mortality in STEMI patients undergoing primary percutaneous coronary intervention.

Unlike previous studies focusing on individual inflammatory markers or isolated outcomes, this study aims to simultaneously evaluate multiple inflammatory indices alongside echocardiographic and angiographic predictors in a contemporary STEMI population undergoing primary PCI.

## 2. Materials and Methods

### 2.1. Study Design and Population

We conducted a retrospective observational study including consecutive adult patients admitted with ST-elevation myocardial infarction (STEMI) and treated with primary percutaneous coronary intervention (PCI) at the Cardiology Department of the Clinical Emergency County Hospital Oradea, Romania, between January 2021 and August 2025. STEMI was defined according to contemporaneous guideline criteria, based on the presence of ischemic symptoms, persistent ST-segment elevation, new left bundle branch block and/or presumed new ischemic electrocardiographic changes, accompanied by elevated cardiac biomarkers.

Inclusion criteria were as follows: (1) age ≥ 18 years; (2) confirmed diagnosis of STEMI according to guideline criteria; (3) treatment with primary PCI as the initial reperfusion strategy; (4) presentation and treatment within the study period; (5) availability of admission laboratory data allowing calculation of inflammatory biomarkers of interest, including C-reactive protein (CRP), absolute neutrophil and lymphocyte counts and platelet count; and (6) documented vital status at hospital discharge.

Exclusion criteria included the following: (1) non-ST-segment elevation acute coronary syndromes or uncertain diagnosis; (2) absence of primary PCI as the reperfusion strategy (thrombolysis only, medical management, coronary artery bypass grafting as first strategy, or no coronary angiography); (3) missing essential laboratory or outcome data preventing calculation of inflammatory indices or determination of hospital outcome; (4) duplicate admissions for the same patient during the study period (only the first admission was retained); (5) active infection at presentation or within the first 24 h; (6) patients with a documented history of chronic heart failure or previously known left ventricular systolic dysfunction (LVEF < 40%) before the index admission (7) chronic kidney disease requiring hemodialysis, chronic severe pulmonary disease, inflammatory or autoimmune disease with active flare or ongoing immunosuppressive therapy; (8) active malignancy, particularly metastatic disease or ongoing oncologic therapy; (9) recent major surgery or trauma within 30 days; (10) hematologic diseases affecting leukocyte or platelet counts; and (11) chronic systemic steroid or immunomodulatory therapy when identifiable from medical records.

A total of 600 patients met the inclusion and exclusion criteria and were included in the final analysis.

### 2.2. Clinical and Demographic Data

Demographic characteristics and cardiovascular risk factors were collected for all patients, including age, sex, smoking status and the presence of diabetes mellitus, arterial hypertension, obesity and dyslipidemia. Time from the first medical contact to first device deployment was recorded as door-to-balloon time, expressed in minutes.

### 2.3. Echocardiographic Assessment

Transthoracic echocardiography was performed during hospitalization according to standard protocols. The following parameters were recorded: left ventricular ejection fraction (LVEF), left ventricular and right ventricular dimensions, left atrial size, presence of moderate-to-severe ischemic mitral regurgitation (grade II–III), moderate-to-severe aortic stenosis, pulmonary hypertension, pericardial effusion and left ventricular thrombus.

### 2.4. Coronary Angiography and PCI Characteristics

Coronary angiography was performed in all patients undergoing primary PCI. Angiographic data included identification of the culprit lesion as well as the presence of significant non-culprit coronary stenoses in the major epicardial vessels. Significant coronary stenosis was defined as ≥70% diameter stenosis in a major epicardial vessel or ≥50% in the left main coronary artery. Information regarding the vessels treated with stent implantation was recorded. Residual coronary disease was defined as the presence of significant non-stented coronary lesions and was expressed as the number of residual non-stented stenoses identified during the index procedure. All patients underwent primary PCI as the initial reperfusion strategy. Patients treated with fibrinolytic therapy before coronary angiography were not included in the study cohort.

### 2.5. Laboratory Measurements

Laboratory parameters, including hemoglobin, serum creatinine and estimated glomerular filtration rate (eGFR), were recorded at admission (within the first hours after hospital presentation) and after PCI. Inflammatory biomarkers measured at admission included C-reactive protein (CRP), neutrophil-to-lymphocyte ratio (NLR), platelet-to-lymphocyte ratio (PLR), systemic immune-inflammation index (SII) and C-reactive protein-to-lymphocyte ratio (CLR). These indices were calculated using standard formulas based on the differential blood count obtained at hospital admission.

### 2.6. Outcome Definition

The primary outcome of the study was in-hospital mortality, defined as all-cause death occurring during the index hospitalization.

### 2.7. Statistical Analysis

Continuous variables were tested for normality using the Shapiro–Wilk test and expressed as mean ± standard deviation or median (interquartile range), as appropriate. Categorical variables were presented as counts and percentages. Comparisons between groups were performed using the Student’s *t*-test or Mann–Whitney U test for continuous variables and the chi-square test or Fisher’s exact test for categorical variables. Correlations were assessed using Pearson or Spearman correlation coefficients, depending on data distribution. Logistic regression analysis was used to identify predictors of in-hospital mortality. Missing data were handled through complete-case analysis. Patients with missing essential laboratory or outcome data, preventing calculation of inflammatory indices or determination of hospital outcome, were excluded according to the predefined exclusion criteria. Consequently, no missing data were present in the final analytical dataset. Model calibration was assessed using the Hosmer–Lemeshow goodness-of-fit test together with Cox–Snell and Nagelkerke pseudo-R^2^ statistics. Multicollinearity among variables included in the multivariable logistic regression model was assessed using variance inflation factors (VIFs). A *p*-value < 0.05 was considered statistically significant. Statistical analyses were conducted using SPSS software version 20.

Candidate variables for multivariable logistic regression were selected based on clinical relevance and the results of univariable analyses. Because of the retrospective nature of the study, several established STEMI prognostic variables, including Killip class, cardiogenic shock at presentation, cardiac arrest before admission, TIMI flow parameters, peak cardiac biomarker levels, and SYNTAX score, were not consistently available and therefore could not be included in the final model.

## 3. Results

### 3.1. Baseline Characteristics According to In-Hospital Mortality

Baseline clinical, demographic and procedural characteristics of the study population stratified according to in-hospital mortality are summarized in Table 1. Of the 600 patients included in the analysis, 54 (9.0%) died during hospitalization, while 546 (91.0%) survived to hospital discharge. Patients who died during hospitalization were significantly older than survivors (71.70 ± 12.39 vs. 61.75 ± 11.58 years, *p* < 0.001). Although female patients exhibited a higher crude in-hospital mortality rate compared with male patients, the association between sex and mortality did not reach statistical significance (*p* = 0.056). With regard to cardiovascular risk factors, diabetes mellitus was significantly more prevalent among non-survivors. In contrast, current smoking and dyslipidemia were significantly less frequent among non-survivors than among survivors (all *p* < 0.01). In contrast, no significant differences were observed in the prevalence of arterial hypertension or obesity between survivors and non-survivors.

Procedural characteristics revealed no significant difference in door-to-balloon time between the two groups (*p* = 0.544). However, patients who died during hospitalization exhibited a significantly higher number of residual non-stented coronary lesions compared with survivors (*p* < 0.001).

Angiographic and PCI characteristics are summarized in Table 2. The left anterior descending artery was the most frequently involved culprit vessel, followed by the right coronary artery and the left circumflex artery. PCI was most commonly performed in the LAD and RCA territories.

### 3.2. Inflammatory Biomarkers and Left Ventricular Dysfunction

Inflammatory biomarkers measured at hospital admission were significantly associated with the severity of left ventricular dysfunction (Table 3). Patients with reduced left ventricular ejection fraction (LVEF ≤ 40%) exhibited significantly higher levels of C-reactive protein (CRP; *p* = 0.002) and C-reactive protein-to-lymphocyte ratio (CLR; *p* = 0.001) compared with patients with preserved systolic function. No significant differences were observed for neutrophil-to-lymphocyte ratio (NLR), platelet-to-lymphocyte ratio (PLR), or systemic immune-inflammation index (SII).

Non-survivors exhibited significantly lower eGFR and higher serum creatinine levels; however, these variables did not remain independently associated with mortality after multivariable adjustment.

Similarly, patients with moderate-to-severe ischemic mitral regurgitation showed a significantly higher inflammatory burden (Table 4). CRP (*p* = 0.018), PLR (*p* = 0.027) and SII (*p* = 0.011) levels were significantly elevated in patients with ischemic mitral regurgitation compared with those without this complication, while NLR demonstrated a borderline association.

No statistically significant associations were identified between inflammatory biomarkers and the presence of moderate-to-severe aortic stenosis, suggesting that systemic inflammatory activation in STEMI is primarily related to acute ischemic myocardial injury rather than pre-existing valvular heart disease.

**Figure 1:**
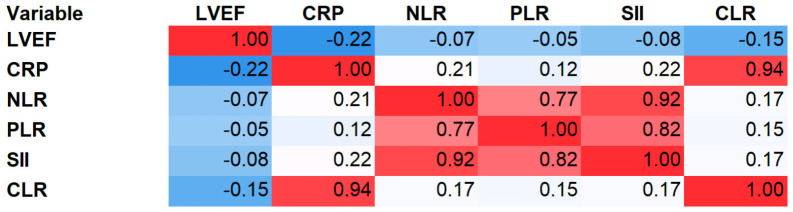
Spearman correlation heatmap between left ventricular ejection fraction (LVEF) and inflammatory biomarkers measured at hospital admission. Color intensity represents the strength and direction of the correlation coefficient (ρ), with blue indicating positive correlations and red indicating negative correlations. LVEF demonstrated significant inverse correlations with C-reactive protein (CRP), C-reactive protein-to-lymphocyte ratio (CLR), and systemic immune-inflammation index (SII), suggesting that higher inflammatory burden was associated with more severe left ventricular systolic dysfunction. Correlation coefficients were calculated using Spearman rank analysis.

Correlation analysis further supported these findings (Figure 1). LVEF was inversely correlated with CRP (Spearman’s rho = −0.22, *p* < 0.001), CLR (rho = −0.15, *p* < 0.001) and SII (rho = −0.08, *p* = 0.039), indicating that higher inflammatory activity was associated with more severe impairment of left ventricular systolic function.

### 3.3. Determinants of In-Hospital Mortality

**Figure 2:**
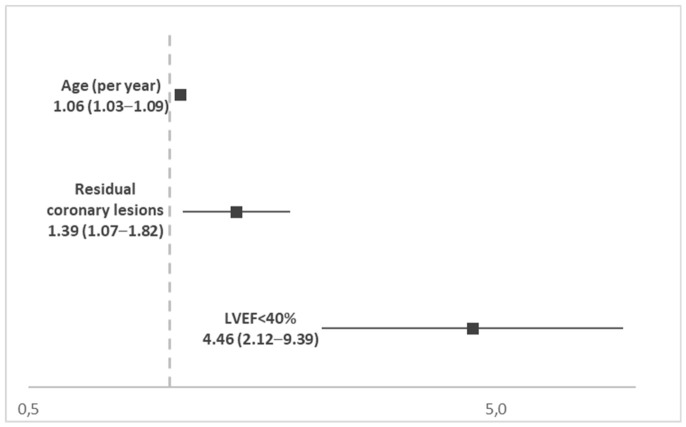
Forest plot showing the association between clinical and echocardiographic variables and in-hospital mortality in the multivariable logistic regression model. Squares represent odds ratios (ORs), while horizontal lines indicate the corresponding 95% confidence intervals (CIs). Variables with confidence intervals not crossing the null value (OR = 1.0) were considered statistically significant independent predictors of in-hospital mortality.

Multivariable logistic regression analysis identified age, left ventricular ejection fraction (LVEF) < 40% and the number of residual coronary lesions as independent predictors of in-hospital mortality (Figure 2). Increasing age was associated with a significantly higher risk of in-hospital death (OR 1.06 per year, 95% CI 1.03–1.09, *p* < 0.001). Patients with LVEF < 40% had a more than fourfold increased risk of mortality compared with those with preserved ventricular function (OR 4.46, 95% CI 2.12–9.39, *p* < 0.001). Additionally, the number of residual coronary lesions was independently associated with mortality (OR 1.39 per lesion, 95% CI 1.07–1.82, *p* = 0.014) (Figure 2). Significant mitral regurgitation and pulmonary hypertension were not found to be significantly associated with in-hospital mortality after adjustment for other covariates. The final multivariable mortality model included five predictors and 54 in-hospital deaths, corresponding to approximately 10.8 events per variable. The model demonstrated acceptable explanatory performance, with a Cox–Snell R^2^ of 0.125 and a Nagelkerke R^2^ of 0.275. Calibration was adequate according to the Hosmer–Lemeshow goodness-of-fit test (χ^2^ = 5.293, *p* = 0.726). No evidence of multicollinearity was observed among predictors included in the multivariable model, with all variance inflation factors below 1.2.

In multivariable logistic regression including CRP, NLR, PLR, SII, and CLR, none of the inflammatory biomarkers were independently associated with in-hospital mortality (all *p* > 0.05). The overall model was not statistically significant (χ^2^ = 6.066, *p* = 0.300) and showed limited explanatory power (Nagelkerke R^2^ = 0.042). Receiver operating characteristic (ROC) analysis further confirmed the limited predictive performance of inflammatory biomarkers (Table 5). All biomarkers demonstrated poor discrimination for in-hospital mortality, with AUC values close to 0.50 and confidence intervals overlapping the null value.

## 4. Discussion

In this study, we examined the interplay between baseline clinical characteristics, inflammatory biomarkers, left ventricular dysfunction and in-hospital mortality in a large cohort of patients presenting with ST-elevation myocardial infarction (STEMI). Our findings demonstrate that advanced age, impaired left ventricular systolic function and a higher burden of residual coronary disease are the main independent determinants of in-hospital mortality. Inflammatory biomarkers may reflect the severity of myocardial injury and ventricular dysfunction; however, because infarct size was not directly quantified, this interpretation should be considered hypothesis-generating.

These findings align with multiple large-scale registries and cohort studies. In a 15-year Japanese registry of 1735 STEMI patients, independent predictors of in-hospital mortality included ejection fraction < 40% (adjusted OR 4.446), Killip class > II (adjusted OR 7.438), chronic kidney disease (adjusted OR 4.056) and culprit lesions in the left coronary artery (adjusted OR 2.940) [18]. Similarly, a multi-ethnic Asian registry of 11,546 STEMI patients identified age, Killip class, cardiac arrest, creatinine and left ventricular ejection fraction as consistent predictors of in-hospital, 30-day and 1-year cardiac mortality [19]. A large U.S. PCI cohort confirmed age, hemodynamic instability, and chronic kidney disease as the strongest predictors of in-hospital mortality [20] Regarding inflammatory biomarkers, although elevated NLR, SII, and CRP are associated with mortality in unadjusted analyses, their independent prognostic value decreases after adjustment for clinical variables such as LVEF, Killip class, and age, suggesting that these biomarkers mainly reflect disease severity rather than providing additional independent prognostic information [21,22].

Patients who died during hospitalization were significantly older than survivors, underscoring age as a powerful determinant of short-term prognosis in STEMI. Advancing age is correlated with a greater prevalence of comorbid conditions, diminished physiological reserve, increased susceptibility to hemodynamic instability and complications during the acute stage of myocardial infarction [23,24,25,26]. Regarding cardiovascular risk factors, diabetes mellitus was significantly more prevalent among non-survivors. Diabetes independently predicts in-hospital mortality, with diabetic patients demonstrating higher rates of cardiogenic shock, more extensive coronary disease and lower revascularization rates [27,28,29,30]. The lower prevalence of smoking and dyslipidemia among non-survivors in our study may reflect the “risk-factor paradox” [31,32,33], likely driven by age-related confounding, as older patients are less often smokers and may have lower documented dyslipidemia. Hypertension and obesity were not associated with in-hospital mortality, suggesting a limited impact on short-term outcomes [34]. Procedural characteristics revealed no significant differences in door-to-balloon time between survivors and non-survivors. While shorter door-to-balloon times are independently associated with lower mortality at the individual patient level, recent population-level improvements in door-to-balloon times have not translated into corresponding mortality reductions, likely due to expansion of PCI to higher-risk patient populations and survivor-cohort effects [35,36,37,38]. In our study, patients who died during hospitalization had a higher number of residual non-stented coronary lesions. This finding highlights the prognostic importance of residual coronary disease burden [39,40,41,42,43,44,45].

Inflammatory biomarkers were associated with the severity of left ventricular dysfunction. Patients with LVEF ≤ 40% showed significantly higher levels of CRP and CLR. [46]. Similarly, patients with moderate-to-severe ischemic mitral regurgitation had higher levels of CRP, PLR and SII, suggesting a possible association between systemic inflammation and ischemic mechanical complications. Greater CRP levels were also independently associated with the presence of moderate or severe mitral regurgitation (*p* < 0.001) and diastolic dysfunction (*p* = 0.002), suggesting that inflammation is related to ventricular remodeling processes independently of LV systolic function [47,48,49]. In contrast, no significant associations were observed between inflammatory biomarkers and moderate-to-severe aortic stenosis, indicating that the inflammatory response in STEMI is primarily driven by acute ischemic myocardial injury rather than pre-existing chronic valvular disease. Correlation analyses confirmed inverse relationships between inflammatory indices (CRP, CLR, SII) and LVEF. Higher SII levels were significantly associated with lower LVEF [50,51,52,53]. Although statistically significant, these correlations were modest, supporting an overall association between higher inflammatory burden and severe LV dysfunction, a key prognostic determinant in STEMI [54,55].

In multivariable analysis, age, left ventricular ejection fraction < 40% and the number of residual coronary lesions were independent predictors of in-hospital mortality. Severely reduced systolic function conferred a more than fourfold increased risk of death, consistent with prior registries reporting adjusted odds ratios of 4.4–4.5 for LVEF < 40% [18,56]. Residual coronary disease also independently predicted mortality, underscoring the importance of overall coronary atherosclerotic burden beyond the culprit lesion [57,58,59]. In contrast, inflammatory biomarkers were not independently associated with in-hospital mortality after adjustment for clinical and angiographic variables, suggesting that their prognostic impact is largely mediated through established determinants such as ventricular dysfunction and coronary disease burden, consistent with prior evidence showing limited prognostic value of inflammatory markers when added to models incorporating clinical and functional variables [60].

Taken together, our findings suggest that while inflammatory biomarkers reflect the severity of myocardial injury and ventricular dysfunction, early prognosis in STEMI is primarily driven by age, left ventricular systolic function, and residual coronary disease. These parameters remain the cornerstone of risk stratification, while inflammatory markers may provide complementary but not standalone prognostic information [15,19,61,62,63].

The present study has several important strengths. First, it comprises a relatively large real-world cohort of consecutive STEMI patients managed in the current era of primary percutaneous coronary intervention, thereby improving the generalizability of the findings. Second, the novelty of this study lies in the integrated assessment of multiple inflammatory biomarkers with left ventricular function and residual coronary disease, allowing direct comparison of their prognostic value in a real-world STEMI cohort. Third, the use of multivariable logistic regression enabled the identification of independent predictors of in-hospital mortality while accounting for relevant confounding factors.

Several limitations should also be acknowledged. The observational and retrospective design precludes the establishment of causal relationships and may be subject to residual confounding. The use of strict exclusion criteria may introduce selection bias and limit the generalizability of the findings to broader real-world STEMI populations. Inflammatory biomarkers were measured at a single time point on admission and therefore, dynamic changes during hospitalization could not be assessed. The relatively small number of in-hospital deaths may have reduced the statistical power to detect weaker independent associations, particularly for inflammatory markers and less frequent complications. Formal internal validation procedures, such as bootstrap resampling or cross-validation, were not performed and therefore the stability of the prediction models could not be formally assessed. In addition, echocardiographic parameters were obtained during the acute phase, when loading conditions and hemodynamic instability may influence measurements.

An additional limitation relates to the availability of established STEMI prognostic variables. Information regarding Killip class, cardiogenic shock at presentation, cardiac arrest before admission, TIMI flow before and after PCI, peak cardiac biomarker levels, and SYNTAX score was not consistently available within the retrospective dataset and therefore could not be incorporated into the multivariable analyses. Consequently, residual confounding cannot be excluded, and the absence of an independent association between inflammatory biomarkers and in-hospital mortality should be interpreted within the context of these unavailable variables.

Finally, long-term follow-up data were not available, preventing the evaluation of the prognostic impact of inflammatory biomarkers on post-discharge outcomes.

## 5. Conclusions

In patients with ST-elevation myocardial infarction, in-hospital mortality is primarily determined by age, left ventricular systolic function, and residual coronary disease burden. Inflammatory biomarkers are associated with the severity of myocardial injury and ventricular dysfunction but do not provide independent prognostic information beyond established clinical predictors. These findings underscore the central role of traditional clinical and angiographic parameters in early risk stratification in STEMI.

## Figures and Tables

**Table 1:** Baseline clinical characteristics according to in-hospital mortality.

Variable	Overall (*n* = 600)	Survivors (*n* = 546)	*Non-Survivors* (*n* = 54)	*p*-Value
**Age, years**	62.65 ± 11.98	61.75 ± 11.58	71.70 ± 12.39	<0.001
**Male sex, *n* (%)**	398 (66.2%)	370 (67.8%)	28 (51.9%)	0.056
**Smoking—current, *n* (%)**	234 (39.0%)	223 (40.8%)	11 (20.4%)	0.003
**Diabetes mellitus, *n* (%)**	181 (30.2%)	156 (28.6%)	25 (46.3%)	0.007
**Hypertension, *n* (%)**	409 (68.2%)	374 (68.5%)	35 (64.8%)	0.579
**eGFR, mL/min/1.73 m^2^**	73.00 (54.00–91.00)	77.00 (57.00–92.00)	50.00 (30.00–64.00)	<0.001
**Dyslipidemia—current, *n* (%)**	189 (31.5%)	181 (33.2%)	8 (14.8%)	0.006
**Obesity, *n* (%)**	60 (10.0%)	56 (10.3%)	4 (7.4%)	0.506
**Door-to-balloon time, min**	101.00 (66.00–230.00)	100 (67.75–230.05)	115.50 (52.00–229.75)	0.544
**Residual non-stented lesions, *n***	0 (0–1)	0 (0–0)	0 (0–2)	<0.001

Data are reported as mean ± standard deviation or median (interquartile range) for continuous variables, and as counts with corresponding percentages for categorical variables. The Shapiro–Wilk test was used to assess normality of distribution. Between-group comparisons were performed using Student’s *t*-test for normally distributed continuous variables, the Mann–Whitney U test for non-normally distributed continuous variables, and either the chi-square test or Fisher’s exact test when appropriate. In-hospital mortality was defined as all-cause mortality occurring during the index hospitalization. A two-sided *p* value of <0.05 was considered statistically significant. Dyslipidemia—current was defined as a documented diagnosis of dyslipidemia at the time of study inclusion.

**Table 2:** Angiographic and percutaneous coronary intervention (PCI) characteristics of the study population.

Coronary Artery	Culprit Lesion Location, *n* (%)	Treated Vessel During PCI, *n* (%)
Left main coronary artery (LM)	20 (3.3%)	10 (1.7%)
Left anterior descending artery (LAD)	379 (63.2%)	335 (55.8%)
Left circumflex artery (LCx)	174 (29.0%)	107 (17.8%)
Right coronary artery (RCA)	316 (52.7%)	245 (40.8%)
Posterior descending artery (PDA)	8 (1.3%)	6 (1.0%)
Obtuse marginal branch (OM1/OM2)	64 (10.7%)	35 (5.8%)
Diagonal branch (D1/D2)	36 (6.0%)	17 (2.8%)

**Table 3:** Inflammatory biomarkers according to left ventricular systolic function.

Biomarker	LVEF > 40%	LVEF ≤ 40%	*p*-Value
**CRP**	12.00 (5.00–37.10)	27.00 (7.22–68.92)	0.002
**NLR**	4.24 (2.59–6.73)	4.96 (3.01–7.40)	0.152
**PLR**	126.45 (87.14–178.22)	132.48 (90.19–192.73)	0.611
**SII**	1003.55 (615.95–1570.50)	1197.27 (625.80–2039.99)	0.102
**CLR**	7.64 (2.68–21.79)	12.33 (3.58–37.96)	0.001

Data are presented as median (interquartile range). Group comparisons were conducted using the Mann–Whitney U test, given the non-normal distribution of inflammatory biomarkers. A two-sided *p*-value < 0.05 was considered statistically significant.

**Table 4:** Inflammatory biomarkers according to the presence of significant mitral regurgitation.

Biomarker	With Significant Mitral Regurgitation (*n* = 76)	Without Significant Mitral Regurgitation (*n* = 524)	*p*-Value
CRP	39.55 (6.75–100.85)	17.00 (5.50–46.30)	0.018
NLR	5.10 (3.23–7.29)	4.45 (2.62–6.88)	0.063
PLR	144.89 (103.53–221.55)	125.71 (86.99–178.64)	0.027
SII	1351.01 (790.24–1919.75)	997.45 (592.81–1860.74)	0.011
CLR	18.55 (4.47–57.44)	8.14 (2.83–24.41)	0.097

Data are expressed as median (interquartile range). Differences between groups were assessed using the Mann–Whitney U test, in light of the non-normal distribution of inflammatory biomarkers. A two-sided *p* value of <0.05 was defined as statistically significant.

**Table 5:** Receiver operating characteristic analysis of inflammatory biomarkers for prediction of in-hospital mortality.

Biomarker	AUC	95% CI	*p*-Value
CRP	0.556	0.437–0.676	0.338
NLR	0.534	0.408–0.660	0.565
PLR	0.494	0.369–0.619	0.918
SII	0.540	0.425–0.655	0.499
CLR	0.525	0.397–0.652	0.676

AUC—area under the curve; CI—confidence interval. Receiver operating characteristic (ROC) analysis was performed to evaluate the ability of admission inflammatory biomarkers to predict in-hospital mortality. None of the evaluated biomarkers demonstrated clinically meaningful discriminative performance.

## Data Availability

The data supporting the findings of this study are not publicly available due to patient privacy and GDPR restrictions.
